# *Clostridioides difficile* in Calves in Central Italy: Prevalence, Molecular Typing, Antimicrobial Susceptibility and Association with Antibiotic Administration

**DOI:** 10.3390/ani11020515

**Published:** 2021-02-16

**Authors:** Francesca Blasi, Carmela Lovito, Elisa Albini, Luca Bano, Gastone Dalmonte, Ilenia Drigo, Carmen Maresca, Francesca Romana Massacci, Serenella Orsini, Sara Primavilla, Eleonora Scoccia, Silvia Tofani, Claudio Forte, Chiara Francesca Magistrali

**Affiliations:** 1 Istituto Zooprofilattico Sperimentale dell’Umbria e delle Marche “Togo Rosati”, 06121 Perugia, Italy; f.blasi@izsum.it (F.B.); e.albini@izsum.it (E.A.); c.maresca@izsum.it (C.M.); fr.massacci@izsum.it (F.R.M.); s.orsini@izsum.it (S.O.); s.primavilla@izsum.it (S.P.); e.scoccia@izsum.it (E.S.); c.magistrali@izsum.it (C.F.M.); 2 Istituto Zooprofilattico Sperimentale del Piemonte, Liguria e Valle d’Aosta, 10154 Torino, Italy; carmela.lovito@izsto.it; 3 Microbiology and Diagnostic Laboratory, Istutito Zooprofilattico Sperimentale delle Venezie, 31020 Villorba di Treviso, Italy; lbano@izsvenezie.it (L.B.); idrigo@izsvenezie.it (I.D.); 4 Istituto Zooprofilattico Sperimentale della Lombardia e dell’Emilia Romagna “Bruno Ubertini”, 25124 Brescia, Italy; g.dalmonte@izsler.it; 5 Istituto Zooprofilattico Sperimentale del Lazio e della Toscana “M. Aleandri”, 00178 Roma, Italy; silvia.tofani@izslt.it; 6 Department of Veterinary Sciences, University of Turin, 10095 Grugliasco (TO), Italy

**Keywords:** *Clostridium difficile*, calves, antibiotics, resistance, ribotypes

## Abstract

**Simple Summary:**

*Clostridioides difficile* is a leading cause of nosocomial and community-acquired diarrhoea in men. The infection most commonly occurs in people who have recently been treated with antibiotics. Indistinguishable *C. difficile* strains have been isolated from livestock and humans, which has shed light on a possible zoonotic origin of this infection. This study aimed to assess the prevalence and risk factors of *C. difficile* in calves bred in dairy and beef cattle farms of the Umbria, central Italy. We estimated a 19.8% prevalence of farms positive for *C. difficile*. The *C. difficile* isolates from calves were potentially toxigenic and resistant to antibiotics, including lincosamides, quinolones, vancomycin and linezolid. Isolates belonging to ribotype RT-126, which is also commonly reported in humans, showed the highest number of resistance to the antimicrobials tested. Furthermore, we observed an almost sixfold increased risk for *C. difficile* on farms where penicillins had been prescribed. This, together with the detection of toxigenic and antibiotic-resistant isolates, strongly suggests the need for a reduction of antibiotic use in cattle.

**Abstract:**

The emergence of *Clostridioides difficile* as the main agent of antibiotic-associated diarrhoea has raised concerns about its potential zoonotic role in different animal species. The use of antimicrobials is a major risk factor for *C. difficile* infection. Here, we provide data on *C. difficile* infection in dairy and beef calves in Umbria, a region in central Italy. This cross-sectional study focuses on prevalence, risk factors, ribotypes, toxinotypes and antimicrobial resistance profiles of circulating ribotypes. A prevalence of 19.8% (CI95%, 12–27.6%) positive farms was estimated, and the prescription of penicillins on the farms was associated with *C. difficile* detection (OR = 5.58). Eleven different ribotypes were found, including the ST11 sublineages RT-126 and -078, which are also commonly reported in humans. Thirteen isolates out of 17 showed resistance to at least one of clindamycin, moxifloxacin, linezolid and vancomycin. Among them, multiple-drug resistance was observed in two isolates, belonging to RT-126. Furthermore, RT-126 isolates were positive for tetracycline resistance determinants, confirming that tetracycline resistance is widespread among ST11 isolates from cattle. The administration of penicillins increased the risk of *C. difficile* in calves: this, together with the recovery of multi-resistant strains, strongly suggests the need for minimising antibiotic misuse on cattle farms.

## 1. Introduction

The Gram-positive, spore-forming, anaerobic bacterium *Clostridioides difficile* (*Clostridium difficile*) is recognised as the main antibiotic agent associated with diarrhoea in human medicine and a major rising cause of gastrointestinal infection in animals [1,2]. *C. difficile* infection in humans (CDI) may cause mild to severe colitis. Pseudomembranous colitis represents the most typical and severe manifestation [3,4].

Historically, CDI has been described as a nosocomial infection, associated with antibiotic treatment and hospitalisation as important risk factors in humans. Similar pathogenesis and risk factors are suggested for animals [5,6]. The rapid changing of epidemiology and the recent increase of CDI in the community placed the attention on *C. difficile* exposure outside the hospital. Observed in humans and animals and in the environment, *C. difficile* is ubiquitous [6]. The potential role of animals as a reservoir for community CDI has been investigated and described in many studies worldwide [7,8,9,10,11].

The epidemiology of *C. difficile* has changed in recent decades with the circulation of new PCR ribotypes with different potential spread both in Europe and worldwide [12]. Recent literature suggests that clones could disseminate internationally with a zoonotic/anthroponotic transmission [10]. For example, a whole-genome phylogenetic analysis revealed a strong connection between humans and animals’ RT-078, linked in a long-range transmission network. Some isolates from humans and veterinary medicine, appear to be identical by using classical and next-generation molecular techniques, showing a genetic overlap of isolates recovered from different animal species [1,13]. In this scenario, animals represent the main suspected source of *C. difficile* for community-acquired infections [14].

It is known that calves and piglets are among the predominant animals harboring *C. difficile*. Newborns in general are characterised by higher prevalence rates since age is a key factor that affects the isolation of *C. difficile* in animals [15]. The consumption of contaminated meat, due to the high prevalence of *C. difficile* in livestock animals (including calves and piglets), represents a potential mechanism for transmission [16].

The aim of this cross-sectional study was to evaluate the prevalence as well as the potential risk factors of *C. difficile* in dairy and beef calves in Umbria, a region situated in central Italy. The study investigated the antimicrobial susceptibility, toxinotypes and ribotypes of the *C. difficile* isolates from calves. Risk factors for the occurrence of *C. difficile*, including the use of antibiotics on farms, were also investigated.

## 2. Materials and Methods

### 2.1. Study Population and Data Collection

A cross-sectional study was conducted on dairy and beef cattle farms of the Umbria region in Central Italy. A total of 101 bovine farms were enrolled between September 2017 and April 2018. The sampling activities were conducted in farms with more than 50 animals, specifically focusing on calf barns, seeing that young age was shown to be a significant risk factor for *C. difficile* shedding in calves [17]. The calf barns sampled hosted calves under 60 days of age. All calves were born on the same farm where they were sampled. The farms included in the study were involved in the animal welfare program of the Umbria region (2014–2020 Rural Development Program of Umbria region, Measure 14. https://www.regione.umbria.it/agricoltura/misura14). 

Of the 101 cattle farms, 47 were dairy farms and 54 were beef farms. Based on the data obtained from the National Database of the National Zootechnical Register, established by the Ministry of Health at the Istituto Zooprofilattico Sperimentale Abruzzo and Molise on the reference date of 31 December 2016 (https://www.vetinfo.it/), the sampled farms represented 40.9% of the farms with at least 50 cattle (101/247) of the region. Variations regarding the herd sizes and livestock category, between the sampled population and the source population, were investigated, highlighting no differences.

The sampling protocol is described in the supplementary materials (File S1) and was drawn up in line with the German antimicrobial resistance program [18]. Briefly, from the calf barns of each farm, four samples were collected as follows: i) three pooled faecal samples of stools on the ground, in close contact with the calves; ii) one pair of boot swabs. A total of 404 samples were collected into sterile containers using sterile gloves, kept at 4 °C and analysed within 12 h. 

Data on antimicrobial consumption, along with the reason for treatments, were collected and analysed as already described by Ferroni et al. [19], from paper-based registers on farms.

### 2.2. C. difficile Culture and Identification

Each sample was homogenised before processing. One gram of each pooled faecal sample was submitted to the *C. difficile* detection method, in accordance with Arroyo et al. [20]. Briefly, the method consists of anaerobic incubation (H_2_–CO_2_–N_2_, 5/5/90%) in 9 mL of cycloserine-cefoxitin fructose broth supplemented with 0.1% sodium taurocholate, TCCFB (*C. difficile* selective supplement, Oxoid, UK; D-(-) Fructose, dibasic Sodium phosphate dihydrate, Potassium phosphate monobasic, Sodium taurocholate hydrate, Neutral red, Sigma-Aldrich, Milan, Italy) for 7–10 days at a temperature of 37 °C. Meanwhile, boot swabs were added to a Stomacher bag (VWR® Blender bag, Leuven, Belgium) containing 45 mL of TCCFB. Subsequently, 10 mL of this suspension was then transferred into sterile tubes anaerobically incubated at 37 °C for 7–10 days.

After incubation, 2 mL of each incubated broth of faecal samples and boot swabs was transferred into a sterile tube containing 2 mL of 96% ethanol for alcohol shock at room temperature for 1 h. The tubes were then centrifuged (3800× *g* for 10 min), and the pellet was streaked on blood agar supplemented with 5% horse red blood cells and 0.1% of esculin or ASEC (Blood Agar base, Biolife Italiana Srl, Milan, Italy; Esculin, Biolife Italiana Srl, Milan, Italy). After anaerobic incubation at 37 °C for 24–48 h, the presence of *C. difficile* was supposed by the smell of a typical horse manure odor. Morphological criteria and the black color (312 nm wavelength) detected through the UV transillumination (TCX-20M, Uvitec, Cambridge, UK) detected suspected colonies, which were isolated on blood agar supplemented with 5% sheep blood (Blood Agar base, Biolife Italiana Srl, Milan, Italy) and incubated anaerobically for 24 h at 37 °C. Characterisation of the species was carried out using polymerase chain reaction (PCR). PCR detected a housekeeping gene (triose phosphate isomerase gene—*tpi*), using conditions described by Lemee et al. [21]. Isolates were stored at −80 °C, and 23 out of 27 isolates were then characterised for ribotype, toxinotype and antimicrobial susceptibility profile. Four isolates were lost in the subculture and storage procedures.

### 2.3. Molecular Characterisation

All the isolates included in this study were screened by PCRs for the presence of the genes encoding toxin *tcdA*, *tcdB*, the binary toxin *cdtA* and *cdtB* and the *tcdC* regulatory gene deletions as previously described [21,22,23]. The characterisation of toxinotypes and ribotypes was performed as described by Rupnik et al. [24] and Bidet et al. [25], respectively. Twenty-nine strains belonging to the PCR-ribotypes circulating in Europe were used as a reference collection (RT-001, RT-002, RT-003, RT-005, RT-010, RT-012, RT-016, RT-017, RT-018, RT-014/020, RT-027, RT-031/1, RT-033, RT-050, RT-056, RT-070, RT-078, RT-081, RT-103, RT-126, RT-127, RT-150, RT-205, RT-403, RT-439, RT-449, RT-548, RT-592, RT-614).

By comparing the profile of the strains included in the study, with the profile of previously captured strains by some research institutes on the base of the ECDC panel, the PCR-ribotypes were identified. Isolates showing an RT pattern different from those reference strains were named using an internal nomenclature (Treviso, TV and number).

The presence of resistance genes *ermB*, *tetM*, *tetO*, *tetL*, *tetS*, *tetK* and *cfr* was assessed, and the isolates were screened by PCRs using primers and protocols previously described [26,27,28].

### 2.4. Antimicrobial Susceptibility Testing

The minimal inhibitory concentration (MIC) for the following molecules amoxicillin-clavulanic acid, ampicillin, clindamycin, erythromycin, linezolid, metronidazole, rifampicin and vancomycin was evaluated using the Etest (Liofilchem, Teramo, Italy), that is, by testing one isolate from each positive farm by simple randomisation. Antimicrobials and the range of concentration tested were in accordance with the work of Thitaram et al. [29]. The rifampicin’s concentration tested was 0.002–32 μg/mL. For the remaining antimicrobials, the range measured was 0.016–256 μg/mL [29]. Briefly, a 1 McFarland standard-matched inoculum was prepared from a 24 h subculture on Brucella blood agar, supplemented with 5% horse red blood cells (Brucella Agar, hemin, Sigma-Aldrich, Milan, Italy) in sterile saline solution. This suspension was then distributed on a Brucella blood agar plate, and Etest strips were applied on the plate. After anaerobic incubation at 37 °C for 48 h, the MIC values were determined according to the manufacturer’s instructions. Furthermore, *Bacteroides fragilis* ATCC 25285 was included as a quality control strain.

The resistance breakpoints for ampicillin, amoxicillin-clavulanic acid, clindamycin, moxifloxacin and metronidazole were derived from CLSI [30]. Regarding rifampicin, vancomycin and linezolid, the breakpoints were set according to the European Committee on Antimicrobial Susceptibility Testing (EUCAST) [31]. Since no clinical breakpoints are available for erythromycin, the MIC values were interpreted by using epidemiological cutoff, and the isolates were classified as wild-type or non-wild-type for erythromycin [https://mic.eucast.org/, accessed on 09 September 2020].

When available, MIC values were compared to the epidemiological cutoff of *C. difficile* reported on the MIC-EUCAST website (https://mic.eucast.org).

According to the clinical cutoffs, an isolate non-susceptible to at least one agent in three or more antimicrobial categories was classified as multi-resistant [32].

### 2.5. Risk Factors

Farms were categorised according to their management and production target in dairy (47) and beef farms (54). The association between *C. difficile* and the following hypothetical risk factors was tested for dairy and beef breeding system, herd size and antimicrobial prescription. Considering herd size, only farms counting more than 50 adult cows were included in the study, as explained in Ferroni et al. [19]. Data on antimicrobial consumption was collected from paper-based registers on farms and analyzed according to Ferroni et al., 2020 [19]. For this purpose, the antimicrobials were grouped into different classes (Table S1).

### 2.6. Statistical Analysis

A descriptive analysis was conducted on data and differences were calculated using Pearson coefficients (Pearson’s χ^2^ test).

The association between some variables and the presence of *C. difficile* has been examined. An odds ratio (OR) and 95% confidence interval (CI95%) were determined for three potential risk factors: different sampling type, dairy and beef production system, and antimicrobial therapy on the farm. The ORs (1) and related 95% confidence intervals (CI95%_OR_; 2) were calculated as follows:

(1) $$\mathrm{OR}=\frac{a\times d}{b\times c}$$

(2) CI95%OR=e ln(OR)±Zα2×1a+1b+1c+1d

They were analysed as categorical variables using univariate analysis.

We tested the association between the variables “herd size” and “breeding system” and the presence of *C. difficile* in different strata of farms. Stratum-specific odds ratio were estimated and the adjusted Mantel-Haenszel estimate of the OR was calculated following the formula reported below:

(3) $${\mathrm{OR}}_{\mathrm{mh}}=\frac{\sum_{i=1}^{s}\frac{a_{i}\;\times {\;d}_{i}}{n_{i}}}{\sum_{i=1}^{s}\frac{b_{i}\;\times {\;c}_{i}}{n_{i}}}$$

*p*-value < 0.05 was considered statistically significant in the chi-square analysis conducted on the data.

Statistical analysis was conducted using Stata software 11.2 (Copyright 2009 Stata Corp LP Stata Corp).

## 3. Results

### 3.1. C. difficile Prevalence and Risk Factors

*C. difficile* was isolated from 27 out of 404 samples (6.7%; CI95%, 4.2–9.1%) and from 20 out of 101 farms (19.8%) (CI95%, 12–27.6%).

**Figure 1:**
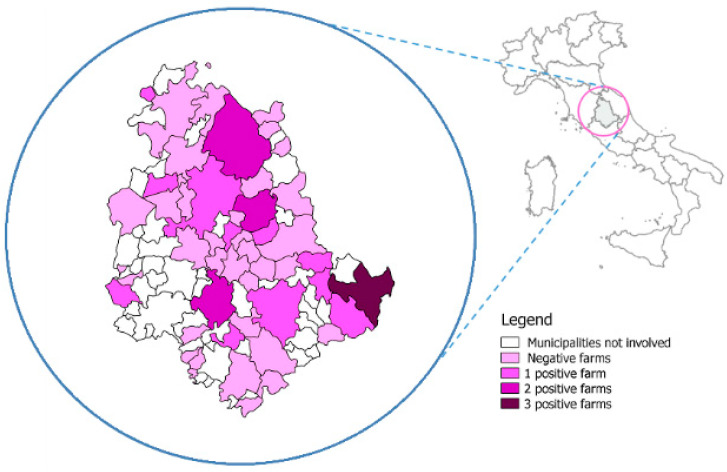
Geographical distribution of *C. difficile* positive farms.

The farms tested were distributed in 47 municipalities in the Umbria region (Figure 1).

The distribution of the different samples types is shown in Table 1. No difference was observed in the proportion of *C. difficile* positive samples between faecal samples and boot swabs (*p* = 0.1447), with a prevalence of 5.6% (CI95%, 3–8.2%) and 9.9% (CI95%, 4.1–15.7%) respectively.

The distribution of the different breeding systems and the herd sizes are shown in Table 2.

No association was found between the farm variables (breeding system and herd size) and the recovery of *C. difficile*. The Mantel–Haenszel summary measure confirmed this lack of association, since the OR (1.21, CI95%: 0.46–3.14) was close to the one without stratification (Mantel–Haenszel test, *p* = 0.6930; Table S2).

The association between the detection of *C. difficile* and antimicrobial prescription on farms is shown in Table S3.

The statistical analysis evidenced a significant association between *C. difficile* isolation and the use of penicillins (*p* = 0.027); more specifically, the use of penicillin on farms increased the odds of being positive for *C. difficile* by almost six times (OR = 5.58) (CI95%, 1.21–25.72).

Accordingly, when the number of treated animals was considered, the prescription of penicillin was associated with the presence of *C. difficile* (*p* = 0.0215) (Table S4). The penicillin prescription includes the association of ampicillin with clavulanic acid.

For the remaining antimicrobial classes prescribed (amphenicols, aminoglycosides, 1ST gen. cephalosporins, 3RD gen. cephalosporins, 4TH gen. cephalosporins, quinolones, fluoroquinolones, lincosamides in association with aminoglycosides, macrolides, rifamycins, sulfonamides and tetracyclines), no significant association with *C. difficile* was observed. Furthermore, there were no associations between the presence of *C. difficile* and the use of antibiotics used in association with another drug.

### 3.2. Ribotypes and Toxinotypes

The characterisation of ribotypes (RT) and toxinotypes were performed on 23 isolates collected from 17 farms. Twelve different PCR-ribotypes were identified, and all of the isolates from the same farm showed the same profile.

As shown in Table 3, RT-033 and RT-078 were detected in three and two dairy farms, respectively. On the contrary, RT-126 was observed in three beef farms. RT-010 was the only ribotype observed in both breeding systems.

### 3.3. Antimicrobial Susceptibility

**Figure 2:**
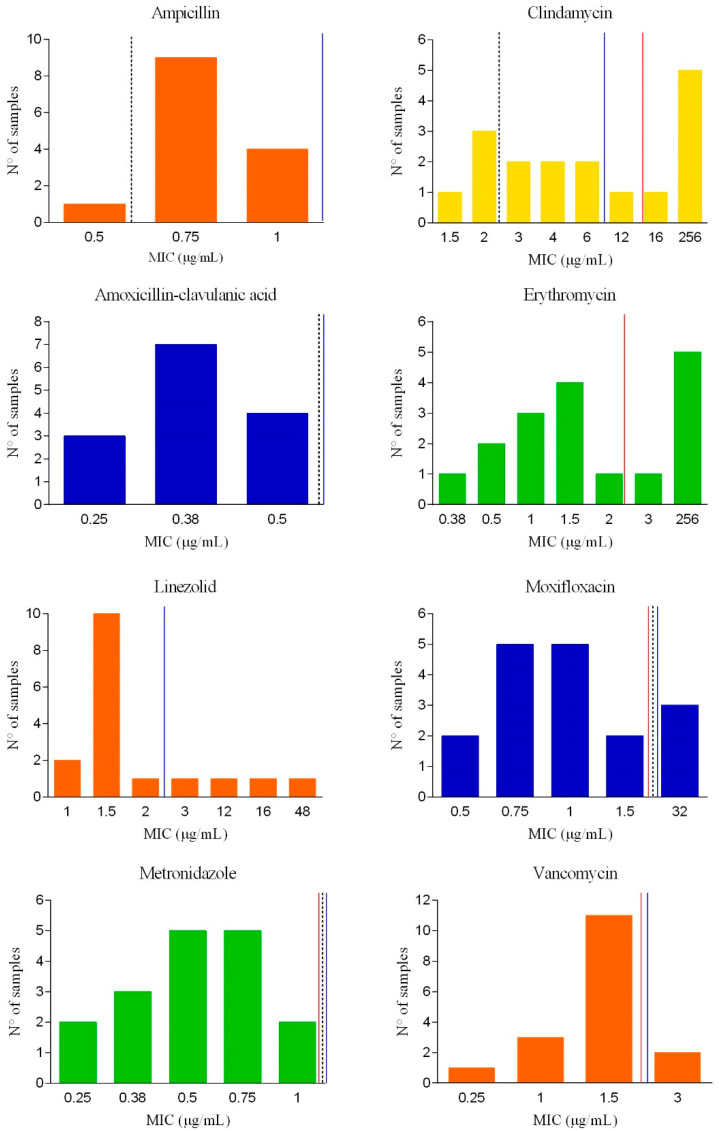
Distribution of minimal inhibitory concentration (MIC) values of *C. difficile* isolates from calves. Clinical breakpoints are represented as dotted lines (representing the limits of full susceptibility) and blue lines (resistance). Epidemiological cutoffs, when available, are represented with red lines.

One isolate per farm was investigated for antimicrobial susceptibility (17 isolates from dairy and beef farms). The MIC values distribution is shown in Figure 2.

All the isolates collected from calves were susceptible to metronidazole and rifampicin. Full susceptibility was observed for ampicillin as well as amoxicillin-clavulanic acid. Thirteen isolates (76.5%) out of 17 displayed a resistance to at least one of the tested molecules. Four out of 17 isolates (23.5%) were resistant to linezolid. Three isolates (17.6%) and two isolates (11.8%) were resistant to moxifloxacin and vancomycin, respectively. Of the 13 clindamycin-resistant isolates (76.5%), five presented high MIC values above 256 µg/mL.

According to the epidemiological cutoff (EUCAST, 2020), six isolates were categorised as non-wild-type (NWT) for clindamycin, and six isolates were classified as NWT for erythromycin.

The isolates were also assessed for the presence of antimicrobial resistance determinants. The *ermB* gene, which confers resistance to macrolide-lincosamide-streptogramin B (MLSB), including clindamycin and erythromycin, was found in only one out of the five isolates, which was resistant to clindamycin and NWT for erythromycin.

The *cfr* gene (multidrug resistance gene) was detected in two out of 17 isolates, which had the following characteristics: they were *ermB*-negative, *tetM*-positive and resistant to clindamycin, linezolid and moxifloxacin. Furthermore, they were both NWT for erythromycin. These two RT-126 isolates also showed the highest number of resistance to the antimicrobials tested and were classified as multi-resistant.

Five isolates out of 17 were positive for tetracycline resistance determinants coding for ribosomal protection protein; specifically, two isolates were positive for *tetM* and three isolates were positive for *tetO*. Additional classes of tetracycline resistance mechanism, such as ribosomal protection protein and efflux pump, were looked for but not found, including those encoded by *tetS*, *tetL* and *tetK*.

The ribotype, toxinotype and antimicrobial susceptibility profile of each isolate are reported in Table 4.

## 4. Discussion

*C. difficile* is a leading cause of antibiotic-associated diarrhoea in humans, and it has been suggested that domestic animals play a role as a reservoir of community-associated infections; particularly for pigs and cattle [1,33,34]. In this study, data on the prevalence of *C. difficile* in calves in Umbria are shown, together with the belonging ribotypes and antimicrobial resistance profiles.

To minimise the sampling-induced stress caused to the animals, in line with the 3Rs principles, the authors decided to use the whole farm and not the single calf as an experimental unit for the study. Moreover, faecal samples were collected from the barn of each herd and not from each calf. As a result, the main limitation of the study is the impossibility to analyse prevalence within the herd as well as the age as the recognised main risk factor [35]. Furthermore, we cannot exclude that recontamination from the farm environment might have occurred. Most of the existing literature focuses on the prevalence of single or few herds, sampling at different ages of the animals [24,36,37,38,39]. Only a few cross-sectional studies are available [40,41,42]. In Italy, this work represents the first prevalence cross-sectional study of *C. difficile* on beef and dairy farms distributed in a limited geographical region.

The farms enrolled in the study were chosen based upon their involvement in an animal welfare program, and this might have caused a selection bias. However, they were distributed across the territory and equally divided between dairy and beef farms. They were characterised only by a few intensive and many semi-intensive farms, as typical for the Umbria region [17]. As reported by Ferroni et al. [19], the sampled population was no different from the regional population in terms of livestock category (beef or dairy) or of herd sizes.

*C. difficile*-positive farms were distributed all over the territory within the Umbria region, but Norcia was the only municipality that had three positive herds. This area was severely affected by an earthquake in 2016–2017; as a result, at the time of sampling, most calves were still hosted in temporary housing, where floors did not allow for the prompt removal of waste. Poor bedding conditions and increased contact of animals with faeces may have facilitated the spread of *C. difficile*, as already suggested in another study conducted on the same species [43].

The observed prevalence in our study (19.8%) is in accordance with prevalence rates in calves reported in the literature worldwide, which have been shown to range from 11.3% [36] to 35.7% [44]. The variability in prevalence estimates observed in the literature may be attributed to host-related factors, such as the age or the breed of the animals. An association between the young age of the animals and *C. difficile* shedding in faeces has been repeatedly reported [17,24,35,36,44,45]. A breed effect was also observed, in terms of a higher prevalence of *C. difficile* in Limousine compared to the Belgian Bleu breed [36]. Differences in *C. difficile* prevalence estimates can also be attributed to the breeding system, even though contradictory findings are reported in the literature. Thitaram et al. [46] found a slightly lower prevalence in beef cattle. In contrast, calves from dairy farms in Germany appeared significantly more likely to harbor *C. difficile* than beef ones [47].

Intensive farming has also been suggested as a possible risk factor, since Bandelj et al. [35] observed a non-linear relation to the number of calves on-farm and *C. difficile* infection. Lastly, the adopted procedures for sample collection and processing can affect the specificity and sensitivity of the test and, consequently, prevalence estimates [46,48,49].

In our study, no difference was recorded between dairy and beef farms. In contrast with the data reported in the literature, the herd size did not influence the prevalence either [35]. This lack of association may be due to the similar breeding system and herd sizes of the farms enrolled in the study. They were mostly family farms of small–medium size and located in a limited area. As an alternative, the number of farms enrolled in this study was not sufficient to detect modest risk factors.

An interesting association was found between the detection of *C. difficile* and penicillin prescriptions on the farm. More specifically, an almost sixfold increased risk of *C. difficile* colonisation was shown in the case of penicillin treatment on the farm. No association was observed for the remaining molecules, probably due to their low utilisation on farms. It should also be noted that we could not link the administration of an antibiotic to an individual animal and the shedding of *C. difficile* from the same calf, and this may have reduced our chances of establishing such an association. In human medicine, penicillins are among the molecules most frequently reported as being associated with *C. difficile* infections [50]. Penicillin administration also appears to increase intestinal exposure to *C. difficile* in horses [51]. Toxigenic *C. difficile* isolates have previously been shown in the caecum of penicillin-treated guinea pigs, although no direct association with penicillin treatment has been observed [52]. In cattle, the role of β-lactams as a risk factor for *C. difficile* had already been observed by the same research group, with a similar odds ratio [17].

In veterinary medicine, the latest data about the sale of antimicrobial agents in Europe indicated tetracyclines and penicillins as the most prescribed classes of antibiotics in food-producing species. The sales account for 32% and 26%, respectively [53]. In Italy, penicillin is largely used in cattle, especially extended-spectrum ones [53].

In our study, RT-126, -033, -078 and -010 turned out to be the most common ribotypes, each isolated from more than one farm. This finding is in accordance with what we have reported in calves in Italy [17] and is consistent with the literature, which indicates that these ribotypes as the most common ones in neonatal calves and piglets in Europe and Asia [15,24,34,40,41,54,55,56,57,58].

The prevalent ribotypes in cattle RT-078 and RT-126 are also frequently isolated in human medicine in Italy [17,59]. Ribotype 010 was not associated with genes coding for virulence factors [60,61] and may be classified as associated with cattle and clinically non-relevant [62]. By contrast, RT-126, 033, 078 belong to clade V, an evolutionary divergent clade of *C. difficile*, which includes both toxigenic and non-toxigenic strains isolated from domestic animals [62]. In this study, isolates RT-126 and RT-078 were classified among the most virulent isolates, being positive for *tcdC* type C gene, *tcdB*, *cdtA* and *cdtB*.

Community-acquired *C. difficile* RT are considered as emerging in CDI in humans. RT-078, the agriculture-associated ribotype, is among the ten most frequent *C. difficile* ribotypes isolated from CDI in western Europe [63]. This ribotype is frequently retrieved from younger people in severe cases of CDI occurring in the community [63], and it is described as the predominant ribotype in pigs, cattle and horses worldwide [64]. RT-078 has also been reported from poultry and dogs, even though in these species it is not considered as the predominant ribotype [62,65,66,67]. The presence of the toxigenic RT-078 has been described in rabbits raised in industrial holdings for food production in Italy [68] and in rats living in urban areas [62]. Finally, RT-078 predominates in wild animals living in direct contact with farms [62]. The importance of environmental contamination in the epidemiology of agriculture-associated ribotypes is confirmed by the detection of RT-078 and RT-126 in bivalves [69].

The prevalence data of RT-126 in humans vary considerably, ranging from 3% in a European survey [70] to 34.4% in Spain [71]. RT-126 has also been reported in other ecological niches, such as river water and different animal species [7,24,72]. In a study conducted in a hospital in central Italy in the same period, RT-126 was also the second most frequently isolated ribotype in cases of CDI [73], thus suggesting a possible common source of such ribotype.

Calves might act as a reservoir for virulent, antibiotic-resistant ribotypes of *C. difficile*. Transmission of *C. difficile* from calves to humans could occur directly, from animals to farmers, as previously reported in pig herds [13,74]. Since toxigenic *C. difficile* have been isolated at harvest and from different meat products post-harvest, the consumption of contaminated meat is another conceivable mechanism for the indirect transmission [41,75,76]. Finally, *C. difficile* isolates shed from calves could contaminate the environment, and spores might survive under adverse environmental conditions. Contaminated water or vegetables may thus represent another CDI indirect transmission pathway [77,78]. It should be noted that in a previous study carried out in Umbria, RT-126 was also recovered from a hospital food (lettuce), thus reinforcing the hypothesis of a transmission from animals to humans through contaminated vegetables [35].

The occurrence of multiple tetracycline-resistant ST11 clones worldwide has supported the hypothesis of a zoonotic origin of these infections, as the use of tetracycline in agriculture is much more common in veterinary than in human medicine [63]. In our study, all the RT-126 isolates carried tetracycline resistance genes, thus confirming that resistance to tetracycline is widespread among ST11 isolates from farm animals.

The multidrug resistance gene *cfr* is common in both Gram-positive and Gram-negative species. In our study, it was detected in the RT-126 isolates, which displayed a non-wild-type phenotype for macrolide and resistance to lincosamide-streptogramin B, thus suggesting the presence of MLSB. Since they were negative to *ermB* determinants, the *cfr* gene could have a significant role in the resistance to MLSB [79].

The ST11 sublineages RT-078 and -126 are frequently reported as resistant to moxifloxacin, clindamycin and erythromycin, and this might provide them with a selective advantage for their dissemination in human and animal populations [9,16]. In our study, resistance to moxifloxacin was detected in two RT-126 and one RT-078 isolates from calves. The two RT-126 isolates were additionally resistant to clindamycin, a combination that favors the emergence of clinically important outbreaks in man [63] and which is particularly frequent in Italy [63,80]. One multi-resistant RT-126 and one clindamycin-resistant RT-078 showed reduced susceptibility to vancomycin, which is the first-choice treatment for moderate to severe cases of CDI. Although nearly all of the *C. difficile* isolates detected in animals in the literature show susceptibility to vancomycin, a small percentage of vancomycin-resistant isolates were already observed in cattle and sheep carcasses [81]. To date, reduced vancomycin susceptibility is rare in men and the few reported cases often belong to the ST11 sublineages RT-078 and -126 [80]. The mechanism of resistance in *C. difficile* is still unclear, representing a potentially serious problem for first-line treatment of CDI [82].

## 5. Conclusions

The role of animals as a reservoir for CDI in humans is being debated. The zoonotic hypothesis is supported by the isolation of indistinguishable strains from different animal species and humans [14].

The use of antibiotics may have a significant impact on the selection and spread of *C. difficile* clones from animals to humans: in particular, resistance to floroquinolones, macrolides, lincosamides and tetracycline has been associated with the spread of ST11 sub-lineages [16]. Thus, our study confirms the occurrence of resistance to these antibiotic classes in *C. difficile* isolates from calves, mostly belonging to ST11.

In addition to inducing a selective pressure towards antibiotic-resistance, the use of antibiotics was also associated with an increased prevalence of *C. difficile* in livestock. Indeed, a nearly sixfold increase has been observed in penicillin prescription, further supporting the need for a reduction of antibiotic use in cattle.

The livestock sector is beginning to move away from the use of antimicrobials. Our data reinforces the need for stewardship programs to further boost this reduction trend.

## Figures and Tables

**Table 1:** *C. difficile* distribution on different types of samples.

Type of Sample	N° of Samples Tested	*C. difficile* Positive Samples (%)	CI95%
Faecal sample	303	17 (5.6%)	3–8.2%
Boot swab	101	10 (9.9%)	4.1–15.7%
Total	404	27 (6.7%)	4.2–9.1%

**Table 2:** *C. difficile* distribution on the two breeding systems and different herd sizes.

Farm Variables	N° of Farms Tested	Positive Farms (%)	CI95%	OR	OR: CI95%	*p*-Value
Breeding system						
Beef farm	54	10 (18.5%)	8.2–28.9%	-	-	-
Dairy farm	47	10 (21.3%)	9.6–33%	1.19	0.45–3.17	0.729
Herd size						
50–99	48	9 (18.8%)	7.3–30.2%	-	-	-
100–199	28	7 (25%)	7.9–42.1%	1.44	0.7–4.43	0.520
≥200	25	4 (16%)	1–31.4%	0.83	0.23–3	0.771

**Table 3:** Molecular characterisation of *C. difficile* isolates from calves (23 isolates from 17 farms).

Ribotype	N° of Positive Farms (%)	Toxinotype	*tcdA*	*tcdB*	Genes Encoding Binary Toxin *CDT*		*tcdC* Gene Deletions ^1^
					*cdtA*	*cdtB*	
RT-126	3 (17.6%)	V/V like	−	+	+	+	−39
RT-078	2 (11.8)	V/V like	−	+	+	+	−39
RT-033	2 (11.8%)	XIa	−	−	+	+	−39
RT-033	1 (5.9%)	XIb	−	−	+	+	−39
RT-010	2 (11.8%)	−	−	−	−	−	N/A
RT-003	1 (5.9%)	0	+	+	−	−	WT
RT-014/020	1 (5.9%)	0	+	+	−	−	WT
RT-449	1 (5.9%)	0	+	+	−	−	WT
TV86 ^2^	2 (11.8%)	0	+	+	−	−	WT
TV87 ^2^	1 (5.9%)	0	+	+	−	−	WT
TV92 ^2^	1 (5.9%)	0	+	+	−	−	WT

^1^*tcdC* gene deletions: (WT) no deletion detected, (−39) deletion of 39 bp, (N/A) not applicable. ^2^ TV stands for internanomenclature (Treviso, TV and number).

**Table 4:** Ribotype, toxinotype and antimicrobial susceptibility data of 17 *C. difficile* isolates, one per farm.

ID Isolate	Ribotype	Toxinotype	Antimicrobials ^1^									Antimicrobial Resistance Genes ^3^
			AMP	AMC	CD	E	MOX	LNZ	MTZ	RD	VA	
A487	RT-078	V/V like	NT	NT	16 *	1	0.75	1.5	0.75	NT	3 *	*tetM*
A489	RT-033	XIa	1	0.25	6	0.5	0.5	1.5	0.25	0.002	1.5	
A490	RT-078	V/V like	0.75	0.25	3	1	32 *	1.5	0.38	0.002	1.5	
A492	RT-033	XIa	NT	NT	12 *	0.5	0.75	1	1	NT	1.5	
A499	RT-010	−	0.75	0.38	256 *	256	1	1.5	0.75	0.002	1.5	
A501	RT-126	V/V like	0.75	0.38	256 *	256	32 *	16 *	0.38	0.002	3 *	*tetM*, *cfr*
A505	RT-010	−	0.75	0.38	256 *	256	1	48 *	0.25	0.002	1.5	
A507	RT-449	0	1	0.5	4	2	0.75	2	0.75	0.002	1	
A510	TV87^2^	0	NT	NT	6	3	0.75	3 *	1	NT	0.25	*tetO*
A511	RT-003	0	0.75	0.38	3	1	1.5	1.5	0.38	0.002	1.5	*ermB*
A512	TV86 ^2^	0	0.75	0.38	2	1.5	1.5	1.5	0.75	0.002	1.5	
A513	TV86 ^2^	0	0.75	0.38	2	1.5	1	1.5	0.5	0.002	1.5	
A514	RT-126	V/V like	0.75	0.5	256 *	256	1	1	0.5	0.002	1.5	*tetO*, *ermB*
A515	RT-014/020	0	0.75	0.5	1.5	1.5	1	1.5	0.75	0.002	1.5	
A516	RT-126	V/V like	1	0.38	256 *	256	32 *	12 *	0.5	0.002	1	*tetM*, *cfr*
A518	RT-033	XIb	0.5	0.25	2	0.38	0.75	1.5	0.5	0.002	1.5	
A519	TV92^2^	−	1	0.5	4	1.5	0.5	1.5	0.5	0.002	1	

^1^ AMP = Ampicillin, AUG = Amoxicillin-clavulanic acid, CD = Clindamycin, E = Erythromycin, LNZ = Linezolid, MOX = Moxifloxacin, MTZ = Metronidazole, RD = Rifampicin, VA = Vancomycin. NT= not tested. ^2^ TV stands for internal nomenclature (Treviso, TV and number). ^3^ *tetM*, *tetO* = tetracycline resistance genes; *ermB* = macrolide-lincosamide-streptogramin resistance gene; *cfr* = phenicol resistance gene. ^*^ Non-susceptible values are in bold and marked with an asterisk.

## Supplementary Materials

The following are available online at https://www.mdpi.com/2076-2615/11/2/515/s1, File S1. Sampling protocol. Table S1. Categorisation of the active principles into the corresponding antimicrobial classes. Table S2. Distribution of farms according to the breeding system and the herd size. The stratum-specific ORs are shown. Table S3. Distribution of antimicrobial classes prescribed on farms and association with *C. difficile*-positive farms. Data on associations of different antimicrobial classes are not shown because they were not significant. Table S4. Descriptive statistics relating to the average number of treated animals for each antibiotic class. Data on associations are not shown.
