# Supplementary material for: Clostridioides difficile in Calves in Central Italy: Prevalence, Molecular Typing, Antimicrobial Susceptibility and Association with Antibiotic Administration
Source: Animals (Basel). 2021 Feb 16;11(2):515. doi: 10.3390/ani11020515 (PMC7920295; doi:10.3390/ani11020515)
Supplement: Supplementary file 1 [file animals-11-00515-s001.pdf]

Supplementary data

File S1. Sampling protocol.
The sampling protocol followed what reported by Hille et al., 2018 [18].
On each farm, the area hosting calves under 60 days of age was divided in three sections: anterior, middle and posterior. Three pooled faecal samples, of at least 10 grams each, were aseptically collected from each of these three sections and immediately put in a sterile container.
The fourth sample consisted of a boot swab sample, which was collected from each calf barn as follows. Boot swabs were commercially available adsorptive polyethylene overboots (Sir Safety System, Assisi, Italy) which were pulled over both feet of the sampling veterinarian. The sampling veterinarian moved across the calf barn, walking along the diagonal of the entire area. On completion of sampling, the boot swabs were carefully removed in order to avoid any losses, inverted to retain of faecal material, and put in a sterile container.
Each sample was individually identified, transported to the laboratory at 4°C and analysed within 12 h from sampling.

Table S1. Categorisation of the active principles into the corresponding antimicrobial classes.

Antimicrobial classes and associations	Active Ingredients (AIs)
**Amphenicols**	
no association	Florfenicol<br>Thiamphenicol
**Aminoglycosides**	
no association	Gentamicin<br>Kanamycin<br>Paromomycin
with Sulfonamides	Paromomycin (+ Dihydrostreptomycin)<br>Neomycin (+ Streptomycin)
**1st gen. Cephalosporins**	
no association	Cefalexin<br>Cefalexin benzathine<br>Cefalonium<br>Cefapirin<br>Cefapirin benzathine<br>Cefazolin
with Aminoglycosides	Cefalexin
with Rifamycins	Cefacetrile
**3rd gen. Cephalosporins**	
no association	Cefoperazone<br>Ceftiofur
**4th gen. Cephalosporins**	
no association	Cefquinome
**Quinolones**	
no association	Flumequine
**Fluoroquinolones**	
no association	Danofloxacin<br>Enrofloxacin<br>Marbofloxacin
**Lincosamides**	
with Aminoglycosides	Lincomycin
**Macrolides**	
no association	Gamithromycin<br>Spiramycin<br>Tilmicosin<br>Tylosin<br>Tulathromycin
with Sulfonamides	Erythromicin

**Penicillins**	
no association	Amoxicillin<br>Ampicillin<br>Procaine benzylpenicillin<br>Cloxacillin<br>Cloxacillin benzathine<br>Penethamate hydriodide<br>Ampicillin (+ Cloxacillin benzathine)<br>Ampicillin (+ Dicloxacillin)
with Clavulanic Acid	Amoxicillin
with Aminoglycosides	Benzylpenicillin<br>Procaine benzylpenicillin<br>Procaine benzylpenicillin (+ Nafcillin)<br>Penethamate hydriodide (+ Benethamine benzylpenicillin)
with Polymyxins	Amoxicillin
**Rifamycine**	
no association	Rifaximin
**Sulfonamides**	
no association	Sulfamethoxypyridazine<br>Sulfadimidine (+ Sulfamerazine + Sulfathiazole)
with Trimethoprim	Sulfadiazine<br>Sulfadimethoxine<br>Sulfadimidine<br>Sulfamonomethoxine
**Tetracyclines**	
no association	Doxycycline<br>Oxytetracycline
with Penicillins	Chlortetracycline

Table S2. Distribution of farms according to the breeding system and the herd size. The stratum-specific ORs are shown.

Breeding system/ herd size	Number of farms	Presence of *C. difficile*	CI95%	OR	P-value
**Beef**					
50-99	29	5 (17.2%)	0%-35.3%	-	-
100-199	15	2 (13.3%)	0%-35.9%	0.74 (0.13-4.35)	1
⩾200	10	3 (30%)	0%-67.3%	2.06 (0.39-10.83)	0.3989
**Milk**					
50-99	19	4 (21.1%)	0%-45%	-	-
100-199	13	5 (38.5%)	3.7%-73.2%	2.34 (0.49-11.26)	0.4269
⩾200	15	1 (6.7%)	0%-23.26%	0.27 (0.02-2.69)	0.3547

Table S3. Distribution of antimicrobial classes prescribed on farms and association with *C. difficile*-positive farms**.** Data on associations of different antimicrobial classes are not shown because they were not significant.

Antimicrobial class prescribed on farm	N° of farms	*C. difficile* positive farms (%)	CI 95%	P-value
**Amphenicols**				0.670
Yes	13	2 (15.4%)	0%-35%	
No	88	18 (20.5%)	12%-28.9%	
**Aminoglycosides**				0.261
Yes	30	8 (26.7%)	10.9%-42.5%	
No	71	12 (16.9%)	8.2%-25.6%	
**1ST gen. Cephalosporins**				0.626
Yes	35	6 (17.1%)	4.7%-29.6%	
No	66	14 (21.2%)	11.4%-31.1%	
**3RD gen. Cephalosporins**				0.727
Yes	37	8 (21.6%)	8.4%-34.9%	
No	64	12 (18.8%)	9.2%-28.3%	
**4TH gen. Cephalosporins**				0.887
Yes	29	6 (20.7%)	6%-35.4%	
No	72	14 (19.4%)	10.3%-28.6%	
**Quinolones**				
Yes	1	0 (0%)		
No	100	20 (20%)	12.2%-27.8%	
**Fluoroquinolones**				0.699
Yes	67	14 (20.9%)	11.2%-30.6%	
No	34	6 (17.7%)	4.8%-30.5%	
**Lincosamides** (with aminoglycosides)				0.940
Yes	31	6 (19.3%)	5.5%-33.3%	
No	70	14 (20%)	10.6%-29.4%	
**Macrolides**				0.396
Yes	42	10 (23.8%)	10.9%-36.7%	
No	59	10 (16.9%)	7.4%-26.5%	
**Penicillins**				**0.027***
Yes	68	18 (26.5%)	16%-37%	
No	33	2 (6.1%)	0%-14.2%	
**Rifamycins**				0.419
Yes	28	7 (25%)	9%-41%	
No	73	13 (17.8%)	9%-26.6%	
**Sulfonamides**				
Yes	5	0 (0%)		
No	96	20 (20.8%)	12.7%-29%	
**Tetracyclines**				0.341
Yes	41	10 (24.4%)	11.3%-37.5%	
No	60	10 (16.7%)	7.2%-26.1%	

Table S4. Descriptive statistics relating to the average number of treated animals for each antibiotic class. Data on associations are not shown.

Antimicrobial class/prescribed on farm	Number of treated animals/100 animals (±SD)	P-value
**Amphenicols**		0.6764
C. difficile positive farms	0.016 (±0.67)	
C. difficile negative farms	0.0123 (±0.0386)	
**Aminoglycosides**		0.116
C. difficile positive farms	0.0714 (±0.1083)	
C. difficile negative farms	0.027 (±0.0594)	
**1ST gen. Cephalosporins**		0.3908
C. difficile positive farms	0.0169 (±0.0824)	
C. difficile negative farms	0.0457 (±0.0823)	
**3RD gen. Cephalosporins**		0.9567
C. difficile positive farms	0.0372 (±0.0986)	
C. difficile negative farms	0.0417 (±0.0908)	
**4TH gen. Cephalosporins**		0.8812
C. difficile positive farms	0.0475 (±0.1253)	
C. difficile negative farms	0.0424 (±0.1505)	
**Quinolones**		0.6193
C. difficile positive farms	0	
C. difficile negative farms	0.0002 (±0.0014)	
**Fluoroquinolones**		0.2015
C. difficile positive farms	0.0806 (±0.0855)	
C. difficile negative farms	0.054 (±0.0873)	
**Lincosamides** (with aminoglycosides)		0.8839
C. difficile positive farms	0.0273 (±0.0619)	
C. difficile negative farms	0.0176 (±0.0412)	
**Macrolides**		0.8415
C. difficile positive farms	0.038 (±0.0646)	
C. difficile negative farms	0.0552 (±0.0957)	
**Penicillins**[1]		**0.0215***
C. difficile positive farms	0.1076 (±0.1439)	
C. difficile negative farms	0.0642 (±0.1069)	
**Rifamycins**		0.2846
C. difficile positive farms	0.0123 (±0.0194)	
C. difficile negative farms	0.0094 (±0.0203)	
**Sulfonamides**		0.257
C. difficile positive farms	0	
C. difficile negative farms	0.0045 (±0.0241)	
**Tetracyclines**		0.4235
C. difficile positive farms	0.0001 (±0.0006)	
C. difficile negative farms	0.0001 (±0.0006)	
